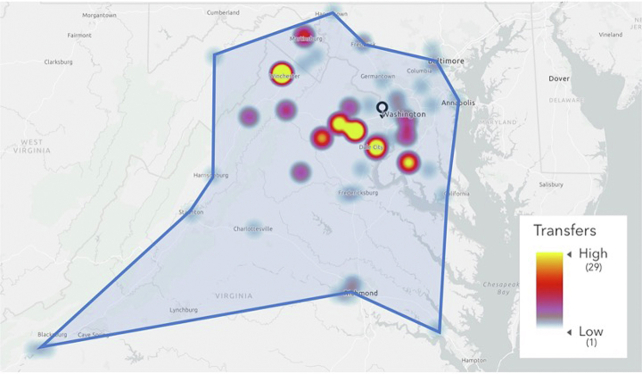# Outcomes of Access Center Transfers for Thoracic Surgical Issues

**DOI:** 10.1016/j.atssr.2025.02.005

**Published:** 2025-03-04

**Authors:** Michael J. Weyant, Abhishek Kumar, Kathryn Bush, Kei Suzuki

**Affiliations:** 1 Department of Surgery, Division of Thoracic Surgery, Inova, Fairfax, Virginia; 2 University of Virginia School of Medicine, Charlottesville, Virginia

## Abstract

**Background:**

Uninterrupted access to thoracic surgical care is limited to large tertiary care centers. Our aim was to characterize the value that interhospital transfers bring to a tertiary care center.

**Methods:**

Retrospective review of access center calls for patients with thoracic surgery needs between April 2022 and August 2023 was performed. Data collected included number/time of calls, diagnosis of requested transfer, number of transfers, number/type of procedures performed, distance of transfer, and characteristics of in-system vs out-of-system patients. Procedures performed were defined as major (performed by thoracic surgeons) or minor (performed by nonthoracic surgery proceduralists).

**Results:**

In total, 367 phone calls occurred over 17 months (22 calls/mo) with 261 calls (71%) leading to transfers. Of those transferred, 169 patients (65%) received an intervention, totaling 218 procedures. Of these procedures, 93 (43%) were major operations, accounting for 15% of thoracic surgery volume, and 125 (57%) were minor procedures. The most common major operations were decortication (33 of 94, 35%), and wedge resections (16 of 94, 17%). Fifty-one out-of-system hospitals accounted for a higher volume (58% vs 42%), and a longer median transfer distance (13.8 vs 48.1 miles). However, in-system transfers were more likely to lead to major thoracic procedures (49% vs 38%).

**Conclusions:**

This is the first study to provide details on interhospital transfers for thoracic surgery needs. A substantial portion of transferred patients undergo an invasive procedure by thoracic surgeons and other proceduralists. The study highlights an underappreciated part of thoracic surgeons’ contribution to patient care in the community and hospital system.

## Visual Abstract

**Figure:**
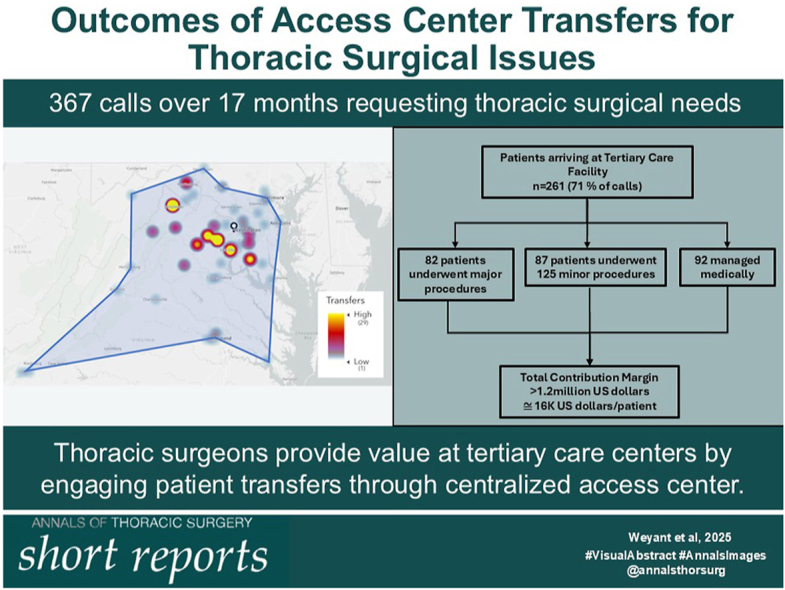

**In Short**

▪ Access center transfers form a notable portion of thoracic surgery practice at a tertial care center.
▪ Thoracic surgery service managing access center transfers provide considerable value to the hospital center.

The system of interhospital transfers is understudied. Although there have been reports characterizing the interhospital transfers for emergency general and vascular surgery patients, mainly focusing on higher morbidity/mortality seen in these patients,^1^, ^2^, ^3^, ^4^ there is a paucity of literature for thoracic surgery. Additionally, there is no literature describing the overall value provided by the presence of thoracic surgical specialists regarding the potential increased revenue and downstream benefits a tertiary care hospital may gain by taking these patients in the form of additional billable procedures and additional care provided by other specialists. Thoracic surgeons do not directly receive all the credit for the downstream revenues created by the consultations we provide as we provide uninterrupted, 24/7 call coverage.

We herein start to better understand how the transfer of thoracic surgery patients impacts both the community as well as the tertiary care center. We also evaluate the value of transferring these patients to the tertiary care center by analyzing procedure yield, contribution margin, and downstream benefit to services other than thoracic surgery.

## Material and Methods

Our hospital is a tertiary hospital system consisting of 5 inpatient hospitals and 4 free-standing urgent care facilities and clinics. The tertiary care facility accepting thoracic surgery transfers is a 930-bed facility.

A retrospective review of access center calls for patients with general thoracic surgery needs, not including cardiac and transplant, between April 2022 and August 2023 was performed. We chose April 2022 as the start date as that is when the access center started collecting objective data. Institutional review board approval was obtained (INOVA-2024-171). Baseline data collected included number/time of calls, insurance type, diagnosis of requested transfer, number of transfers, number/types of procedures performed, distance of transfer, and characteristics of in-system vs out-of-system patients. The number of imaging studies was also recorded. Hospital geographic types were characterized based on population (metropolitan ≥50,000, micropolitan 10,000-50,000, and rural <10,000). Procedures performed were defined as major (operations performed by a thoracic surgeon) or minor (those being performed by nonthoracic surgery proceduralists).

Data regarding contribution margin for the major thoracic cases performed were collected. Hospital contribution margin was calculated by the hospital finance team and reflected all actual revenues less all actual direct costs and estimated indirect costs. Cost of transfer was not accounted for as these were paid by the patients. Direct costs include cases without reimbursement and/or collection. Hospital contribution margin is expressed as 2023 US dollars.^5^

## Results

### Characteristics of Calls, Transfers, and Referring Hospitals

**Figure 1:**
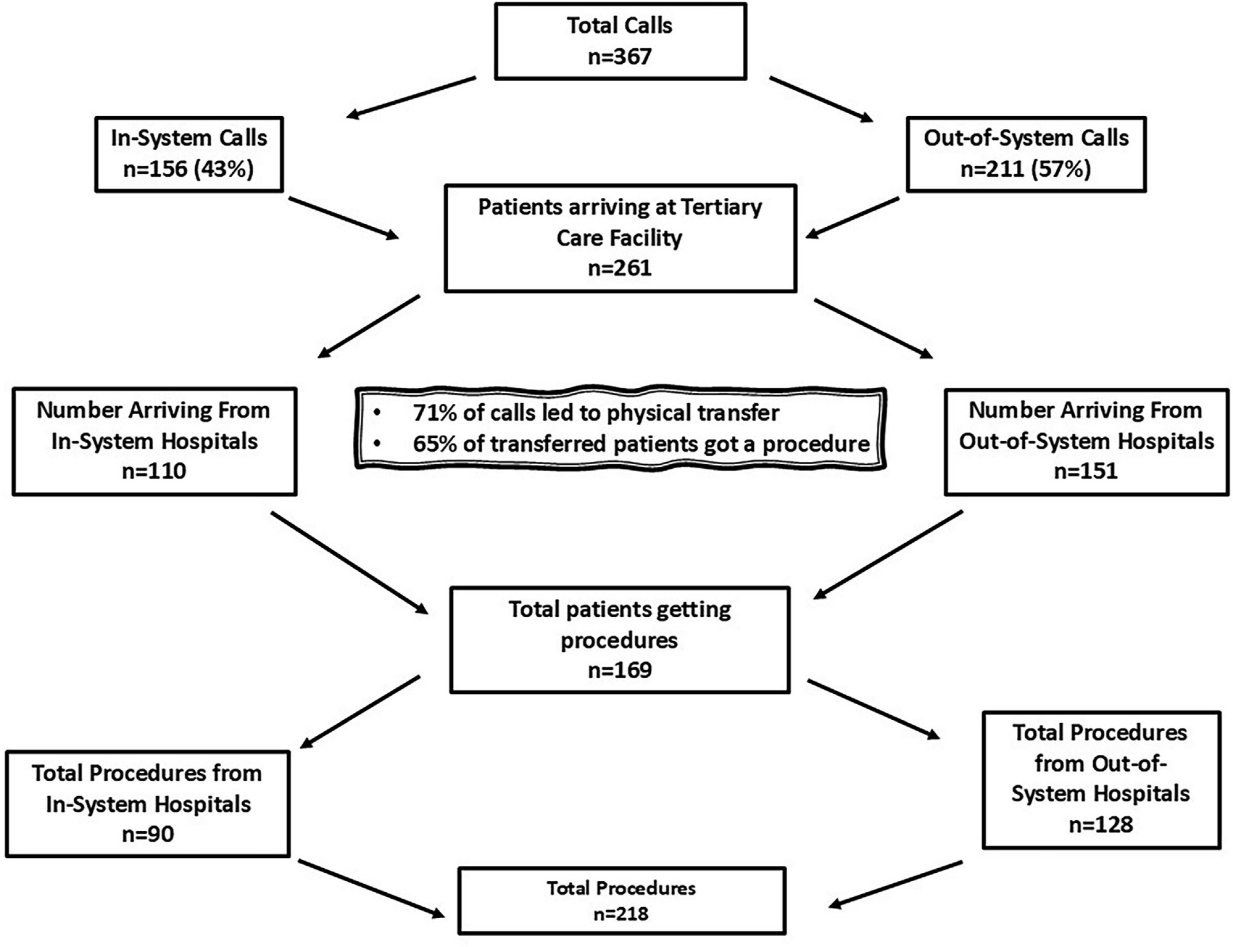
Summary of total calls received by access center at a tertiary care center including total procedure yield.

Patients were transferred from 8 in-system facilities and 51 out-of-system hospitals. In total, 367 transfer requests occurred over 17 months (22 calls/mo) with 261 (71%) calls leading to transfers (Figure 1). For 106 (29%) calls, based on the conversations with the requesting provider and the on-call thoracic surgeon, the transfer was deemed unnecessary. More patients were transferred from out-of-system hospitals (n = 151, 58%) than in-system hospitals (n = 110, 42%). Most common services requesting transfer were emergency medicine (115, 53.4%), internal medicine (50, 23.5%), and family medicine (14, 6.6%).

The geographic catchment area was approximately 21,000 mi^2^, representing a population of approximately 8 million people (Supplemental Figure). The mean distance of transfer was 42.7 miles (range, 1-259 miles). In-system hospitals had a median transfer distance of 13.5 miles (range, 1-28.3 miles) compared with out-of-system transfers having a longer median transfer distance of 65.8 miles (range, 8.7-259 miles). Among the 51 out-of-system hospitals, the median number of beds was 182.5. Seventeen (33%) were in metropolitan, 32 (63%) were in micropolitan, and 11 (22%) were in rural settings.

### Characteristics of Procedures

**Figure 2:**
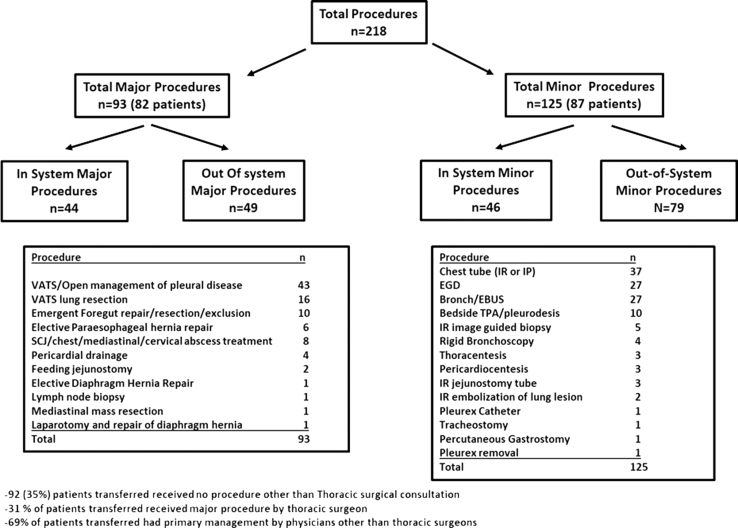
Total procedures resulting from patient transfers into tertiary care hospital. (Bronch, flexible bronchoscopy; EBUS, endobronchial ultrasound; EGD, esophgaogastroduodenoscopy; IP, interventional pulmonology; IR, interventional radiology; SCJ, sternoclavicular joint; TPA, tissue plasminogen activator; VATS, video assisted thoracoscopic surgery.)

The total number of patients receiving any procedure was 169 (65%), with a total of 218 procedures being performed (Figure 1). Ninety-three major procedures were performed on 82 (31%) patients who were transferred (22% of total transfers; Figure 2). One hundred twenty-five minor procedures were performed on 87 (33%) patients (24% of total transfers). Although the 51 out-of-system hospitals accounted for a higher volume of transferred patients (58% vs 42%), in-system transfers were more likely to lead to major thoracic procedures (49% vs 38%; Figure 2).

Major operations were most commonly performed for management of pleural space disease (n = 43, 46%), diagnostic or therapeutic lung resection (n = 16, 20%), and emergency foregut cases (n = 10, 11%) (Figure 2). Of the 10 emergency foregut cases, 5 were for hiatal hernia with possible volvulus/incarceration, and 5 were for esophageal perforations. The most common minor procedures included pleural drainage (n = 37, 30%), esophagogastroduodenoscopy (n = 27, 22%), and bronchoscopy (n = 27, 22%) (Figure 2). Major operations accounted for 13% (82 of 631) of thoracic surgery patient volume during the study period. Among those receiving major operations, major complications included 3 deaths, 2 returns to the operating room, and 2 transfers to higher level of care. Other complications included 4 prolonged air leak, 5 readmissions, and 2 contained leaks, one of which required a feeding tube and another managed conservatively. Among minor procedures, most commonly performed procedure was chest tube (37), followed by upper endoscopy (27), and bronchoscopy/endobronchial ultrasound (27).

Ninety-two (35%) patients transferred received no other procedure other than thoracic surgery consultation. Eighty-one (69%) patients transferred had primary management or procedure by physicians other than thoracic surgeons. Excluding imaging related to procedures, the transfers led to 2278 imaging studies (8.7 studies/patient). One hundred-fifty of the calls (41%) took place between 5 pm and 6 am. One hundred six calls (29%) that did not result in transfer stayed at the referring facility after reassurance.

### Insurance Status and Contribution Margin

**Figure 3:**
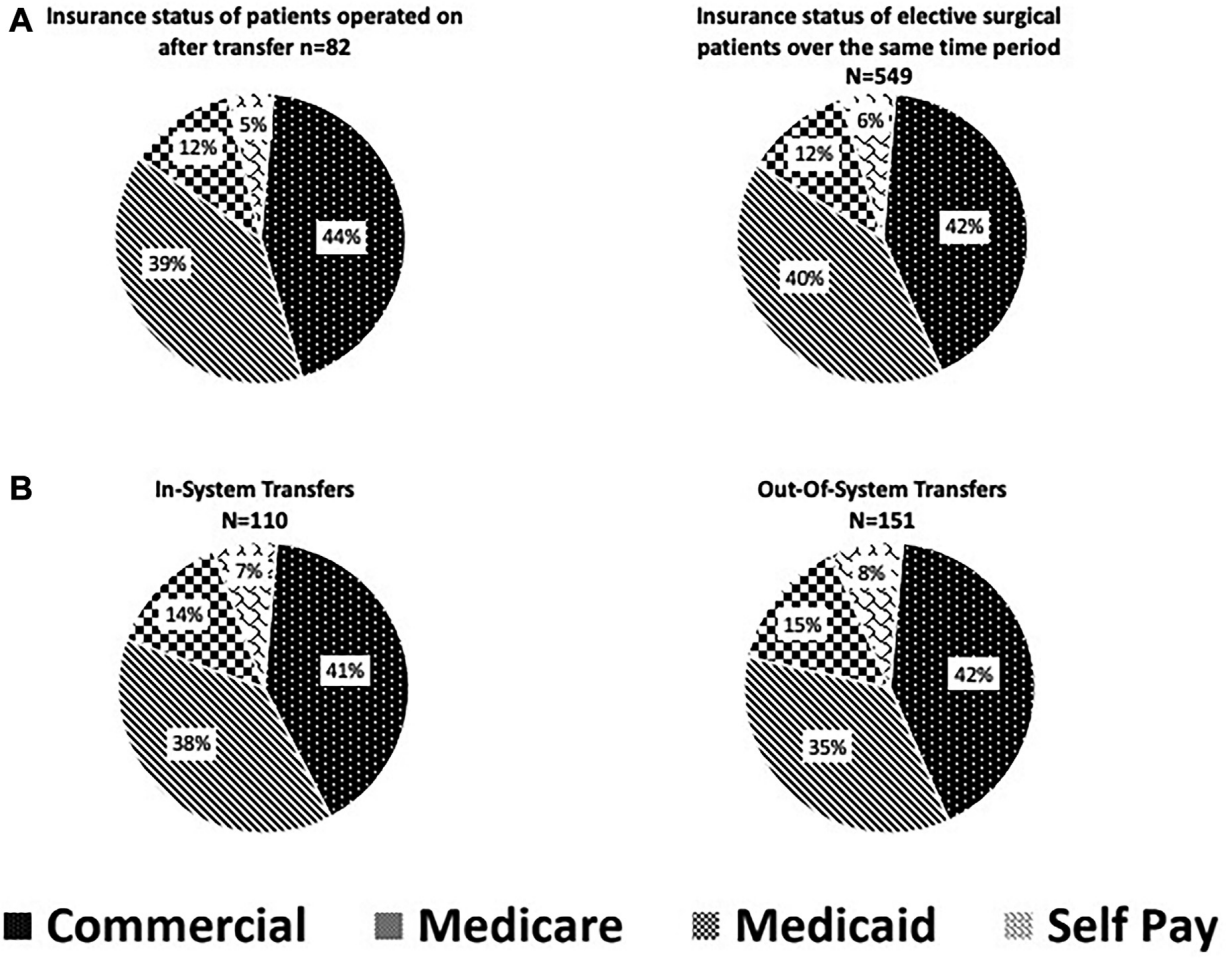
(A) Representation of payor mix of patients undergoing surgery after transfer compared with all other electively scheduled cases. (B) Representation of payor mix of patients transferred from within one of the system hospitals compared with those transferred from hospitals outside the system.

In comparison with all other electively scheduled thoracic surgery procedures during the same study period, there is no difference in the makeup of the payor mix of either population (Figure 3A). Additionally, a comparison of the payor mix of in-system transfers compared to out-of-system transfers showed a nearly identical payor mix in both groups (Figure 3B).

The total contribution margin (79 of 82 patients) related to major thoracic procedures performed as a result of transfer was $1,264,576 ($892,461/y; Table). Average contribution margin per patient was $15,807 ± $23,460. Patients having an isolated decortication procedure (n = 22) as a result of transfer had an average contribution margin of $21,492/patient. Patients undergoing emergent foregut procedures had an average contribution margin of $23,210. By insurance, average contribution margin was $29,321 for commercial insurance, $2397 for Medicare, $23,265 for Medicaid, and $9829 for self-pay.

**Table:** Financial Impact of Transferring Patients for Thoracic Surgical Needs

Financial Impact	Value, $
Total contribution margin	1,264,576
Range of individual case contribution margin	28,360-79,495
Average contribution margin/operation ± standard deviation	15,807 ± 23,460
Average annual contribution margin	892,461

Hospital contribution margin was calculated by the hospital finance team and reflected all actual revenues less all actual direct costs and estimated indirect costs. Direct costs include cases without reimbursement and/or collection.

## Comment

In the current study, we investigated the practice patterns from patients transferred for thoracic surgery consultation to better understand our practices and to understand the benefit and the contribution margin to the hospital. Our data show that a substantial portion (71%) of the requests for transfer appeared necessary, with an appreciable number of patients ultimately needing a major operation. The limitations of this study are its retrospective nature and single institution sourcing; however, given that this report is the first of its kind there is value in using this material as a platform to conceptualize further studies.

The value of thoracic surgical specialists at tertiary care centers is often solely assessed by collections and revenue from professional fees or standardized to a predetermined level of relative value units produced. The complexity of patients who are treated by thoracic surgeons in tertiary care centers is often underestimated and is not factored into the value provided by specialized surgeons. Furthermore, thoracic surgeons provide intangible value in providing support and backup for potential complications that may be encountered by many other procedural specialties such as interventional pulmonology, interventional gastroenterology, and interventional radiology. A much less commonly discussed form of determining value is by analyzing contribution margin to the hospital. Resnick and associates^6^ illustrated that contribution margins of thoracic surgeons can be near the highest of any surgical specialty, even eclipsing cardiac surgery and transplant surgery. We show here that performing just 82 procedures on transferred patients led to a contribution margin of greater than $1.2 million. We also found that certain subgroups of procedures (decortication $23,210/patient, emergency foregut procedures $23,210/patient) led to notable contribution margins. In comparison, elective lobectomies, whose average length of stay is one day, average $11,748/patient, and coronary artery bypass grafting average $31,000/patient.

Contribution margin is not the only value metric we identify in this study. We show that having a thoracic surgeon interact with an access center is also a driver of business toward other procedural specialties. We show that the majority of these nonsurgical procedures are driven toward interventional pulmonology, interventional radiology, and gastroenterology. With these data we can infer that a tremendous service is being provided to the community with the ability to direct the patient to the proper specialist to receive the most advanced care.

The operations performed here represent 13% of our larger thoracic surgery practice and the cases performed on transfer patients are a much different mix than the makeup of the majority of our complex thoracic oncology cases. Despite this, the cases performed on transfer patients require considerable time, often happen at inconvenient times, and require a significant level of skill to perform safely. Illustrating the significant contribution margin is only a small part of quantifying the true effort involved in performing these cases and should be a factor in determining how many trained surgeons are needed to fulfill the requirements of handling these transfer patients. The current relative value unit and professional fee model is an entirely inadequate measure of effort for these patients.

We analyzed insurance status of patients to understand whether there exists a difference in payor mix in this group of patients. Broman and colleagues^7^ suggested that in patients being transferred for emergency general surgery problems, patients with less favorable insurance were more likely deemed candidates for emergency transfer to a higher level of care. Our data suggested that there was no difference in the payor mix of patients transferred for thoracic surgical care.

Our data show that a meaningful number of requests (29%) for access do not lead to transfers and similarly 35% of patients transferred received no additional procedures and had care by a primary medical team with subspeciality consultation. The requests for access that do not lead to transfer are largely unavoidable. However, this subset of patients represents an unquantified risk of providing opinions without all pertinent patient data other than the conversation with the referring provider. This suggests that more work should be done to understand what resources should be used so that more educated opinions can be provided in real time. Southard and coworkers^8^ reported that the creation of a specialized team of an administrator and medical staff helped to facilitate transfers. Similarly, the 35% of patients who were transferred and did not receive a procedure of any kind should be analyzed to understand if further savings can be achieved by avoiding transfer.

In conclusion, this is the first study to provide details on access center calls to a tertiary care center with uninterrupted coverage by specialty thoracic surgical care. Specialty thoracic surgical care provides a notable benefit to the surrounding community and downstream hospital services. An appreciable portion of transferred patients undergo an invasive procedure by thoracic surgeons and other proceduralists. Out-of-system transfers constitute a large source of transfers, indicating a benefit for the larger medical community. Furthermore, the major operations performed as a result of transfer offer a meaningful and previously undescribed source of contribution margin for the tertiary care center. Importantly, 35% of transferred patients underwent no additional intervention and this represents an opportunity for marked cost savings if these transfers could be prevented. The study highlights an underappreciated part of thoracic surgeons’ value and contribution to patient care in the community and hospital system. These factors need to be considered when assessing the true value of thoracic surgical specialists as well as the size of staff needed to support this care.

## Figures and Tables

**Supplementary Figure 1:**