# Supplementary material for: Outcomes of Access Center Transfers for Thoracic Surgical Issues
Source: Ann Thorac Surg Short Rep. 2025 Mar 4;3(3):822–7. doi: 10.1016/j.atssr.2025.02.005 (PMC12559610; doi:10.1016/j.atssr.2025.02.005)
Supplement: Supplementary Figure Legend [file mmc1.docx]

**SUPPLEMENTARY FIGURE LEGEND**

**Supplementary Figure 1**. Geographic heatmap demonstrating catchment area in blue, which represents an area of approximately 21,000 mi2 and a population of approximately 8 million people.
